# Supplementary material for: Advantages of Unfair Quantum Ground-State Sampling
Source: Sci Rep. 2017 Apr 21;7:1044. doi: 10.1038/s41598-017-01096-6 (PMC5430793; doi:10.1038/s41598-017-01096-6)
Supplement: Supplementary file 1 — Supplementary Information [file 41598_2017_1096_MOESM1_ESM.pdf]

Advantages of Unfair Quantum Ground-State Sampling

Brian Hu Zhang[1,+], Gene Wagenbreth[2,+], Victor Martin-Mayor[3,4,+], and Itay Hen[5,6,+,*]

[1]Stanford University, Stanford, California 94305, USA
[2]Cray, Seattle, WA 98164, USA
[3]Departamento de Física Teórica I, Universidad Complutense, 28040 Madrid, Spain
[4]Instituto de Biocomputación y Física de Sistemas Complejos (BIFI), Zaragoza, Spain
[5]Information Sciences Institute, University of Southern California, Marina del Rey, California 90292, USA
[6]Department of Physics and Astronomy and Center for Quantum Information Science & Technology, University of Southern California, Los Angeles, California 90089, USA
[+]these authors contributed equally to this work
[*]itayhen@isi.edu

Supplementary Information

1 The D-Wave Two Quantum Annealer

The D-Wave Two (DW2) processor is marketed by D-Wave Systems Inc. as a quantum annealer. The annealer evolves a physical system of superconducting flux qubits according to the time-dependent Hamiltonian

$$H(t) = A(t)\sum_i H_d^{\mathrm{TF}} + B(t)H_p, \quad t \in [0, \mathscr{T}]\,. \tag{1}$$

The problem Hamiltonian H_p is given by

$$H_p = \sum_{\langle ij \rangle} J_{ij}\sigma_i^z\sigma_j^z + \sum_i h_i\sigma_i^z\,, \tag{2}$$

where σ_i^z is the Pauli Z spin-1/2 matrix acting on spin i, the set $\{J_{ij}\}$ are programmable parameters and $\langle ij \rangle$ sums over the edges of an $N = 504$-qubit Chimera graph—the hardware graph of the processor. The transverse field driver Hamiltonian H_d^{TF} is given by

$$H_d^{\mathrm{TF}} = -\sum_i \sigma_i^x\,, \tag{3}$$

where σ_i^x is the Pauli X spin-1/2 matrix acting on spin i. The annealing schedules given by $A(s)$ and $B(s)$ are shown in Fig. 1 as a function of the dimensionless parameter $s = t/\mathscr{T}$. Our experiments used the DW2 device housed at University of Southern California's Information Sciences Institute, which is held at an operating temperature of 17mK. The Chimera graph of the DW2 used in our work is shown in Fig. 2. Each unit cell is a balanced $K_{4,4}$ bipartite graph. In the ideal Chimera graph (of 512 qubits) the degree of each vertex is 6 (except for the corner cells). In the actual DW2 device, only 504 qubits are functional.

2 Constraint Solver Algorithm

This is a description of the algorithm used to search for and list all solutions, or minimizing configurations, of a spin glass instance with a planted solution. The algorithm takes as input a specified set of constraints, namely, sets of bit assignments, each of which minimizes the local Ising Hamiltonians' H_j, where the total planted-solution Hamiltonian is $H = \sum_j H_j$. Each such term is minimized by a finite set of spin configurations (involving only those spins on which the local Hamiltonian is defined). The algorithm is an exhaustive search and is an implementation of the bucket elimination algorithm described in Ref.[1].

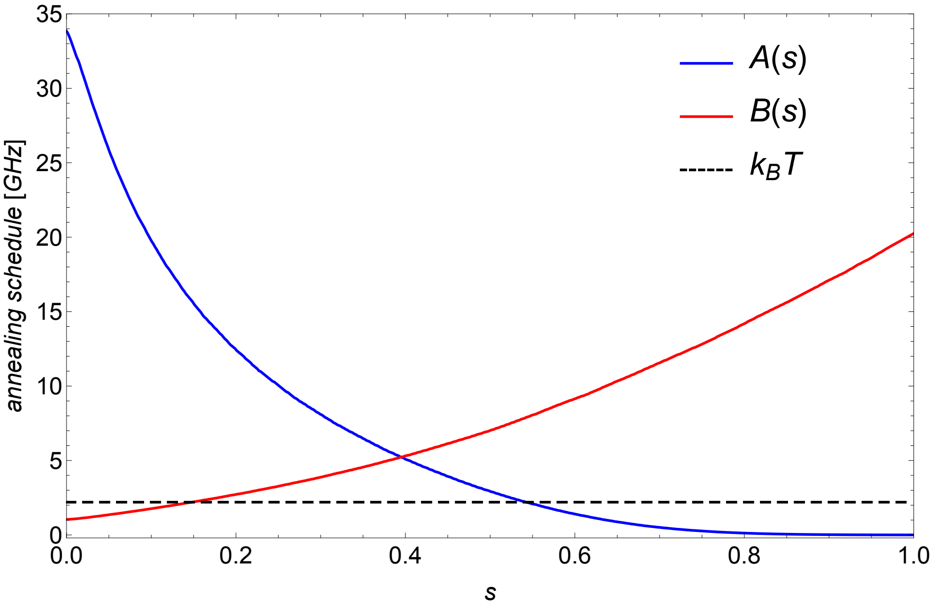

Figure 1. Annealing schedule of the DW2. The annealing curves $A(s)$ and $B(s)$ with $s = t/\mathscr{T}$ are calculated using rf-SQUID models with independently calibrated qubit parameters. Units of $\hbar = 1$. The operating temperature of 17mK is also shown as a dashed line.

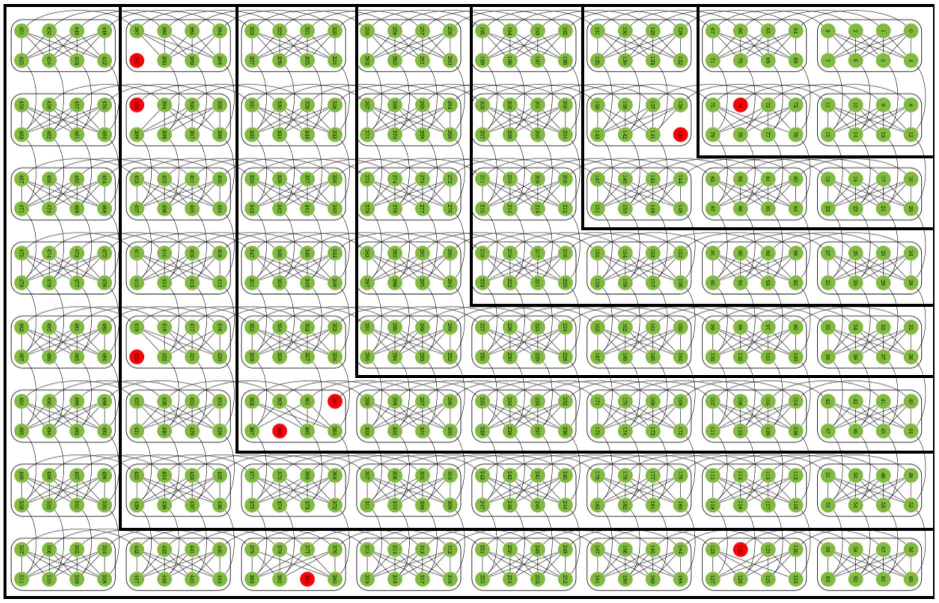

Figure 2. The DW2 Chimera graph. The qubits or spin variables occupy the vertices (circles) and the couplings J_{ij} are along the edges. Of the 512 qubits, 504 were operative in our experiments (green) and 8 were not (red).

The problem structure consists of a set of Ising spins (equivalently, bits) where each value may be $+1$ or -1. A set of constraints restricts subsets of the bits to certain values. The goal is to assign values to all the bits so as to satisfy all the constraints. Each constraint (in this case, the optimizing configurations of the H_j Hamiltonians) applies to a subset of the bits. Each constraint contains a list of allowed settings for that subset of bits. The constraint is met if the values of the bits in the subset matches one of the allowed settings. The constraints for a particular problem are read from a text file. A sample input for a single constraint is:

bit index	sol. #1	sol. #2	sol. #3	sol. #4	sol. #5	sol. #6	sol. #7	sol. #8	sol. #9	sol. #10	sol. #11	sol. #12
480	1	-1	1	-1	1	-1	1	-1	1	-1	1	-1
485	-1	1	1	-1	1	-1	1	-1	1	-1	1	-1
493	1	-1	1	-1	-1	1	-1	1	-1	1	-1	1
491	-1	1	-1	1	-1	1	1	-1	1	-1	1	-1
495	1	-1	1	-1	1	-1	1	-1	-1	1	-1	1
487	1	-1	1	-1	1	-1	1	-1	1	-1	-1	1

The first column is the bit numbers, six in total. The following columns are allowed values for this subset of bits indicating that the term has 12 minimizing bit assignments. This input specifies 12 allowed settings for bits $480, 485, 493, 491, 495$ and 487.

The bucket elimination algorithm, as applied to this problem, consists of the following steps:

```
First, determine if solutions exist:
  while(true)
    select a bit to eliminate
      if no more bits:
        exit loop
      save constraints which contain selected bit
      combine all constraints containing bit
      generate new constraints without bit
      if combined constraints have contradiction:
        exit loop
  end while
```

This algorithm is guaranteed to find all solutions, but may exceed time and memory limitations. All steps are well-defined except for the step which selects the next bit to eliminate. The order in which bits are eliminated dramatically affects the time and memory required. Determination of the optimal order to eliminate bits is known to be NP complete.

Assume there exists a list of constraints, as described above, each of which contains a specified bit. For simplicity, first assume there are only two such constraints. For example, consider the following two simple constraints, both of which contain bit #1:

constraint #1:	bits	#1	#2	#3
allowed settings:	(1_a)	-1	-1	-1
	(1_b)	+1	+1	-1
constraint #2:	bits	#1	#2	#4
allowed settings:	(2_a)	-1	-1	-1
	(2_b)	+1	-1	-1

To combine the two constraints, combine each of the allowed settings in the first constraint with each of the allowed settings in the second constraint. When combining two settings, any bit that is in both settings must agree, or no new constraint is generated. The bit chosen for elimination is not contained in the newly generated constraint. If at the end, no new constraints have been generated, a contradiction exists which prevents these constraints from mutual satisfaction. This indicates that the original problem has no solutions.

Application of this step to constraints $1_a, 1_b, 2_a$ and 2_b above requires four steps. The bits contained in the newly created constraints contain the union of the bits in the constraints, minus bit #1, the bit being eliminated.

	combined configuration
combine 1_a and 2_a	$(-1, -1, -1)$
combine 1_a and 2_b	empty – bit #1 differs in the two
combine 1_b and 2_a	empty – bit #1 differs in the two
combine 1_b and 2_b	empty – bit #2 differs in the two

The new constraint consists of a single entry for bits #2, #3, and #4 set to $(-1,-1,-1)$. To combine more than two constraints, the first two are combined, then the result is combined with the next constraint and repeated until all constraints have been combined. The process of combining constraints can cause the number of allowed bit set values to shrink, as in the example above, or to grow.

The amount of time and memory required to eliminate all bits from the original set of constraints is highly dependent on the order in which bits are eliminated from the original set of constraints. Many heuristics were tested to select the best next bit to be eliminated. No deterministic algorithm was found that yielded acceptable time and memory use on all of the input data sets. The approach that was ultimately found to be effective was to use a combination of heuristics and randomness to select the next bit. Each time an elimination bit is to be chosen, one of six heuristics is chosen at random. The six heuristics are different functions of the number of unique bits in the constraints to be combined, the maximum number of solution sets in any of the constraints to be combined, and the sum of the number of solution sets in the constraints to be combined.

Solutions are enumerated by iterating over the saved variable tables in reverse order. The last table contains the allowed values for the last variable eliminated. Each of these values can be substituted into the previous table to generate allowed value sets for the last two variables. This process is repeated for each table until allowed value sets for all variables are generated. The list is truncated at each step if the number of value sets exceeds a specified limit.

References

1. Dechter, R. Bucket elimination: a unifying framework for processing hard and soft constraints. *Constraints* **2**, 51–55 (1997). URL http://dx.doi.org/10.1023/A:1009796922698.
